# ﻿Notes on the genus *Syzygium* (Myrtaceae) from Cambodia, Thailand, Vietnam, China and Taiwan

**DOI:** 10.3897/phytokeys.244.118657

**Published:** 2024-07-11

**Authors:** Pranom Chantaranothai

**Affiliations:** 1 Applied Taxonomic Research Center (ATRC) and Centre of Excellence on Biodiversity (BDC), Department of Biology, Faculty of Science, Khon Kaen University, Khon Kaen 40002, Thailand Khon Kaen University Khon Kaen Thailand; 2 Royal Botanic Gardens, Kew, Richmond, Surrey TW9 3AE, UK Royal Botanic Gardens Richmond United Kingdom

**Keywords:** Lectotype, Myrtales, new record, new species, synonym, taxonomy

## Abstract

*Syzygiumkampotense* is a new species from Cambodia. *Syzygiumcerasiforme*, *S.foxworthianum*, and *S.angkae* and *S.thorelii* are new records from Cambodia, Vietnam and China, respectively. Syzygiumprainianumsubsp.minor and S.densinerviumvar.insulare are placed under *S.prainianum* and *S.densinervium*, respectively. *Eugeniacochinchinensis*, *E.eburnea* and *E.ripicola*, are reduced to synonymy under *S.pellucidum*. Lectotypes for Eugeniadensifloravar.angustifolia, *E.pellucida* and E.pellucidavar.contracta are designated.

## ﻿Introduction

*Syzygium* Gaertn. is the largest genus in the family Myrtaceae and many species from allied genera, namely *Acmena* DC. and *Cleistocalyx* Blume, are now included in *Syzygium* based on molecular studies. Thus *Syzygium* currently comprises ca. 1,200−1,500 species (Biffin et al. 2006; Craven and Biffin 2010), the majority of which occur in the Old World tropics and subtropics (Biffin et al. 2006). A comprehensive revision of the genus in Thailand was published by Parnell and Chantaranothai (2002). Since then, the number of taxa recorded in Thailand has increased through the discovery of new species and new country records (Chantaranothai 2014; Soh and Parnell 2015; Chantaranothai et al. 2016; Tagane et al. 2018). Similar discoveries have been made in China (Chen and Craven 2007) and Indochina (Soh and Parnell 2015).

During my visits to herbaria in Asia and Europe between 2017 and 2023, many unidentified specimens of *Syzygium* from Thailand and neighbouring countries were examined. Material of an unidentified taxon from Cambodia was found to represent a species new to science, which is described below. Previously unidentified specimens of *S.cerasiforme* (Blume) Merr. & L.M.Perry from Cambodia, *S.foxworthianum* (Ridl.) Merr. & L.M.Perry from Vietnam, *S.angkae* (Craib) Chantar. & J.Parn. and *S.thorelii* (Gagnep.) Merr. & L.M.Perry from China are newly recorded. Syzygiumprainianum(King)Chantar. & J.Parn.subsp.minor Chantar. & J.Parn. and S.densinervium(Merr.)Merr.var.insulare C.E.Chang are placed under *S.prianianum* and *S.densinervium*, respectively. *Eugeniacochinchinensis* Gagnep., *E.eburnea* Gagnep. and *E.ripicola* Craib (*S.ripicola* (Craib) Merr. & L.M.Perry) are reduced to synonymy under *S.pellucidum* (Duthie) N.P.Balakr. Lectotypes are designated for E.densiflora(Blume)Duthievar.angustifolia Ridl., *E.pellucida* Duthie and E.pellucidavar.contracta Wall. ex Duthie.

## ﻿Material and methods

This study is based on both herbarium and field collections in Thailand. Herbarium material was also consulted in the following herbaria: AAU, BK, BKF, BM, K, KKU, KYO, P and QBG (herbarium acronyms following Thiers, updated continuously). Specimens were examined with a binocular microscope and via digital images on the JSTOR website (https://plants.jstor.org/). An illustration of the new species was prepared.

## ﻿Taxonomic treatment

### ﻿ Syzygium kampotense

Taxon classificationPlantaeMyrtalesMyrtaceae

Chantar. sp. nov.

5367B493-4AE2-57B3-A634-B9744FD39D48

urn:lsid:ipni.org:names:77345020-1

Figs 1 , 2 , 3

#### Diagnosis.

**Figure 1.**
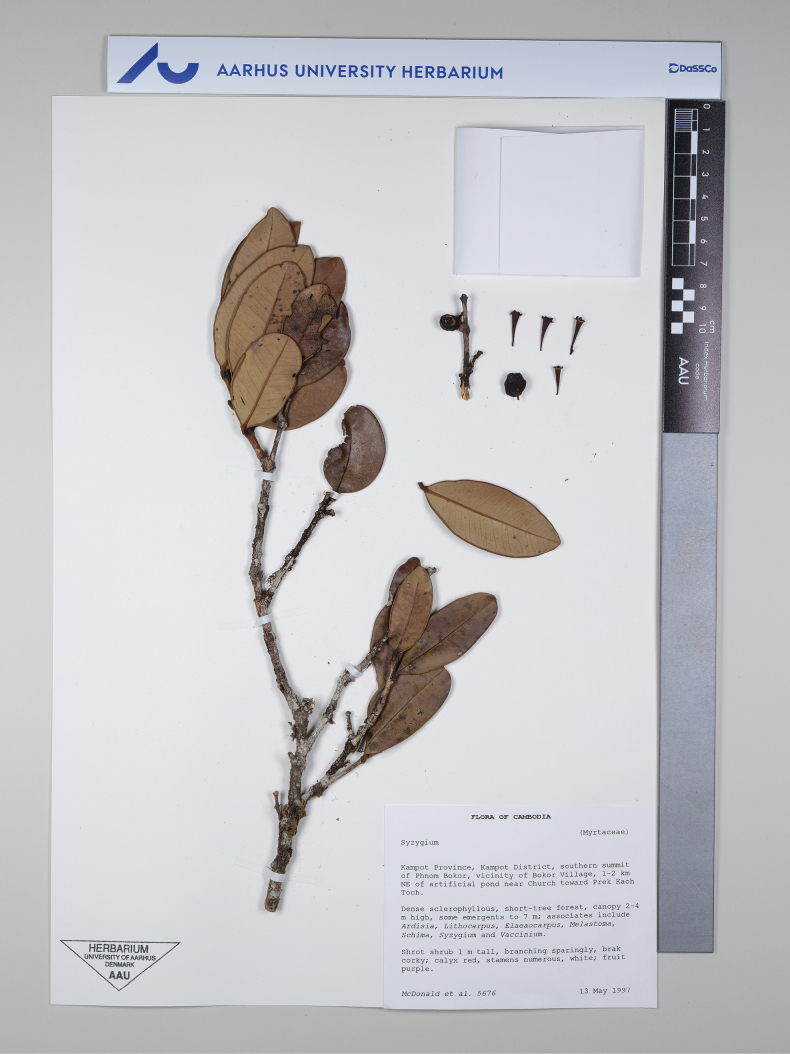
*Syzygiumkampotense* Chantar.: holotype, *McDonal et al. 5676* (AAU). In set leaf, hypanthial cups and fruit. Photographed by B. Boonsuk.

*Syzygiumkampotense* resembles *S.championii* (Benth.) Merr. & L.M.Perry and *S.claviflorum* (Roxb.) Wall. ex Steud., in the clavate hypanthium shape, cuneate leaf base, small leaves and short petioles. The new species differs from both species in having thickly coriaceous leaves with strongly revolute leaf margins, an acute or obtuse leaf apex and fewer secondary veins (Fig. 1, Table 1).

**Table 1.** Morphological comparison of *S.kampotense* and similar species.

Characters	*S.championii*	*S.claviflorum*	*S.kampotense*
Petiole (mm)	1.5–2	3–6	2–3
Leaf texture	coriaceous	chartaceous to coriaceous	thickly coriaceous
Leaf shape	elliptic to oblong	elliptic, ovate, sometimes obovate	elliptic or elliptic-oblong
Leaf size (cm)	4–8.5 × 1–4	6–10 (–22) × 1.5–3.5(–7.5)	4–6.5 × 1.7–3
Leaf base	cuneate, slightly attenuate	cuneate, slightly attenuate	broadly cuneate
Leaf apex	acuminate with acumen	mostly acute without acumen, sometimes acuminate with distinct acumen	acute or obtuse
Leaf margin	flat	flat	strongly revolute
Secondary veins (pairs)	25–30	15–30	ca 12
Hypanthium cup (mm)	10–13	5–10	13–15
Sepal (mm)	0.5 × 2	0.5–1 × 0.8–2.5	ca. 1 × 1.5
Fruit (mm)	oblongoid, clavate, 12.5 × 5	ellipsoid to obovoid, 1–15 × 5–9	globose or ellipsoid, 8–10 × 8–10

#### Type.

Cambodia: Kampot Province, Kampot District, southern summit of Phnom Bokor, vicinity of Bokor, 1–2 km NE of artificial on near Church toward Prek Kaoh Toch, 13 May 1997, *McDonal et al. 5676* (holotype AAU, isotype QBG).

**Figure 2.**
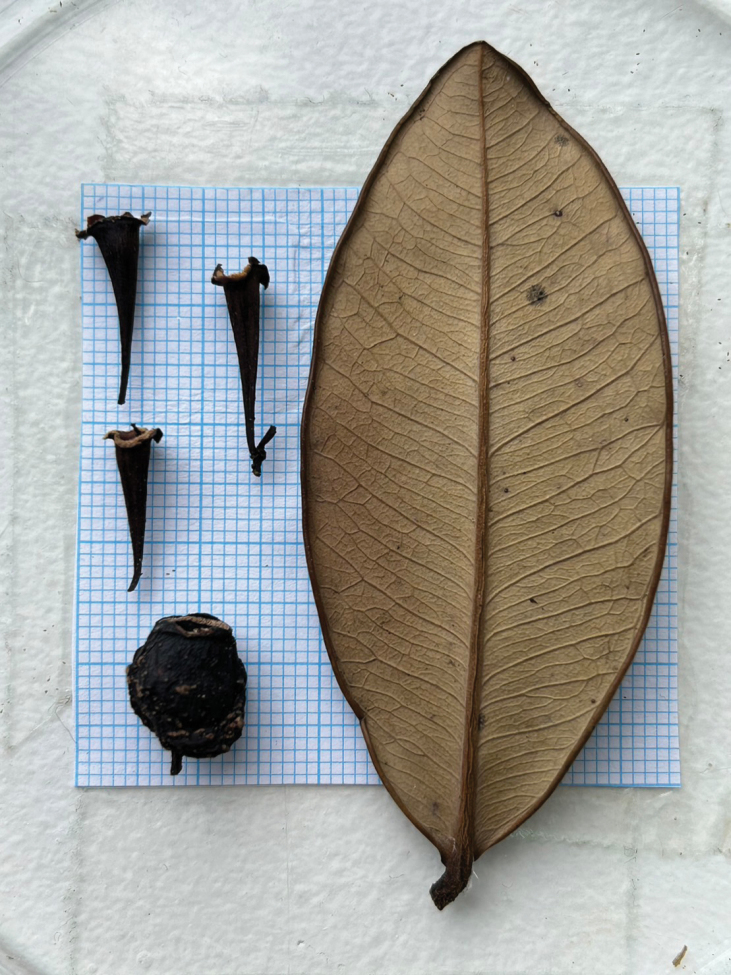
*Syzygiumkampotense* Chantar., showing leaf, hypanthial cups and fruit. Photographed by B. Boonsuk.

#### Description.

Shrub ca. 1 m tall; branching sparingly, bark corky, whitish grey or reddish. ***Leaves*** with petiole 2–3 mm long, wrinkled; lamina thickly coriaceous, 4–6.5 × 1.7– 3 cm, elliptic or elliptic-oblong, base broadly cuneate, apex acute or obtuse, margin strongly revolute; midrib impressed on the upper surface, rounded with sparse pustules on the lower surface; secondary veins in ca. 12 pairs, indistinct on upper surface and distinct on lower surface; intramarginal vein 1. ***Inflorescence*** not seen. ***Hypanthial cup*** 13–15 mm long, clavate. ***Pseudostipe*** absent. ***Sepals*** red 4, ca. 1 × 1.5 mm, triangular. ***Petals*** not seen. ***Stamens*** numerous, white. ***Ovary*** 2-locular, ca. 9 ovules per locule. ***Fruit*** purple, 8–10 mm in diameter, globose or ellipsoid, crowned with remnant of calyx lobes.

**Figure 3.**
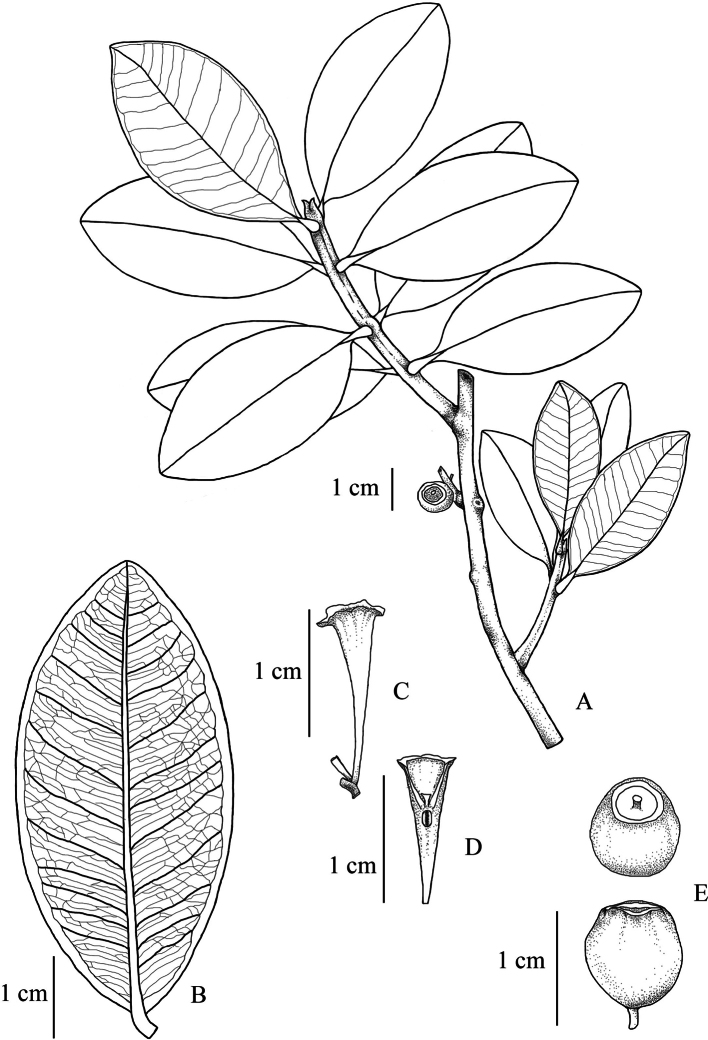
*Syzygiumkampotense***A** habit **B** lower surface of leaf **C** calyx tube (side view) **D** longitudinal section of calyx tube and ovary, showing two locules **E** fruit (from *McDonald et al. 5676*; drawn by N. Triyutthachai).

#### Distribution.

Endemic to Cambodia.

#### Ecology.

Dense sclerophyllous, short-tree forest, canopy 2–4 m tall, some emergent to 7 m tall; associates include *Ardisia*, *Lithocarpus*, *Elaeocarpus*, *Melastoma*, *Schima*, *Syzygium* and *Vaccinum*.

#### Conservation status.

The species is only known from the type locality. It should be categorised as Critically endangered [CR, B1ab (iii)] according to the IUCN Red List Criteria and Categories version 3.1 (IUCN 2012). The Extent of Occurrence is estimated to be less than 20 km^2^ and this species is found in a restricted area on open limestone hills which is a threatened ecosystem.

#### Etymology.

The name of this species is given based on the location where the plant was collected.

#### Notes.

The new species most likely belongs to SyzygiumsubgenusPerikion Craven & Biffin (Craven and Biffin 2010; Hatt et al. 2023, because of its clavate hypanthium shape and ellipsoid, obovoid or globose fruit. These characteristics resemble *S.claviflorum*, the type of this subgenus.

#### Additional specimens examined.

Cambodia, Kampot Province, Bokor National Park, near field station, near top of plateau, 10°20.38'N, 104°01.4'E, alt. 1,045 m, 10 Mar. 2001, *Midlleton & Monyrak 660* (P [P00589160]); Bokor, *Dy Phon 1130* (P [P04658853]).

##### ﻿New Records

### ﻿ Syzygium cerasiforme

Taxon classificationPlantaeMyrtalesMyrtaceae

(Blume) Merr. & L.M.Perry, Mem. Amer. Cad. Arts. 18: 187. 1939.

5D11985B-4DE8-5DCA-8D53-2EDA32BD918F

Myrtus cerasiformis Blume, Bijdr. Fl. Ind. Ned.: 1087. 1826. Type: Indonesia, Java, Blume s.n. (Isotype NY [NY00405548]).

#### Type.

Based on *Myrtuscerasiformis* Blume.

#### Distribution.

Thailand, Cambodia, Malaysia, Java, Borneo.

#### Ecology.

In evergreen swamp forest and a drainage along river.

#### Notes.

POWO (2023) accepts *Syzygiumlineatum* as a synonym of *S.cerasiforme* and also shows the distribution of *S.cerasiforme* in Cambodia, Laos, Vietnam and China. I agree with Merrill and Perry (1939) that both species are not conspecific. Moreover, Parnell and Chantaranothai (2002), Chen and Craven (2007), and especially Soh and Parnell (2015) and Tagane et al. (2015, 2018) working on Cambodian taxa, indicated that *Syzygiumcerasiforme* had never been recorded in Cambodia. *Syzygiumcerasiforme* is similar to *S.lineatum* in having terminal and axillary inflorescences, leaf shape and 14 or more pairs of secondary veins. It differs by smaller leaves, sepals and petals and the outer stamens and style are shorter (4.3–6.7 mm vs 10–15 mm and 5.2–7.7 mm vs 11–13 mm, respectively). However, the Cambodian specimens were collected from Kampong Thom and Stung Treng provinces in Cambodia and I identified them as *S.cerasiforme*. Therefore, these two specimens are the first new record for Cambodia.

#### Specimens examined.

Cambodia, Kampong Thom Province, Sandan District, Rey Long, ca. 13 km south-eastern of Spong, ‘Cheum Takong’ (Takong swam; 3 × 4 km), a drainage of O Long River, Base camp 13°20.27'N, 105°36.077'E, 7 Apr. 2008, *McDonald et al. 7901* (AAU) & Stung Treng Province, Prey Long Forest, Cheum Takong and O Long River, 13°20.359'N, 105°36.32'E, 7–10 April 2009, *McDonald et al. PL8* (AAU).

### ﻿ Syzygium foxworthianum

Taxon classificationPlantaeMyrtalesMyrtaceae

(Ridl.) Merr. & L.M.Perry, Mem. Mer. Acad. Arts 18: 168. 1939.

8D75CC25-F2C2-53FA-9150-41EF97574BB1

Eugenia foxworthyi Ridl., Fl. Mal. Penins. 1: 728. 1922, non Elmer, 1912.
Eugenia foxworthiana Ridl., Fl. Mal. Penins. 5: 308. 1925. Type: Peninsular Malaysia, Pahang, Bukit Goh Reserve, 12 Jan. 1920, *Foxworthy Field No. 3624* (lectotype, designated by Chantaranothai and Parnell 1994, p. 63: K [K001005521]).
Syzygium foxworthianum (Ridl.) Masam., Enum. Phan. Born.: 528. 1942.
Eugenia densiflora Miq. var. angustifolia Ridl., Fl. Mal. Penins. 1: 729. 1922. Type: Peninsular Malaysia, Perak, Ulu Temengoh, Ridley s.n. (lectotype, designated here: K [without barcode]).
Syzygium pycnanthum Merr. & L.M.Perry var. angustifolium (Ridl.) P.S.Ashton, Tree Fl Sabah & Sarawak 7: 270. 2011.

#### Type.

Based on *Eugenafoxworthiana* Ridl.

#### Distribution.

Thailand, Peninsular Malaysia, Sumatra, Borneo, Vietnam (Lao Cai (Laokhay), Pakha, 10 Dec. 1935, *Poilane 25052* (K).

#### Ecology.

In evergreen forest, 50–200 m alt.

#### Notes.

Ashton (2011) placed *S.foxworthianum* under *S.pycnanthum* (*Eugeniadensiflora* Miq.) but I do not agree with this placement because the former differs from the latter by having a long and lax inflorescence (vs short and dense), slightly smaller and narrower leaves, 16–18.5 × 4–6.5 cm (vs larger and broader, 17.5–26 × 4.5–8.5 cm), two intramarginal veins, sometimes without the intramarginal vein and secondary veins ascending to a shallow loop (vs 2–3 intramarginal veins). Although the Vietnamese specimen *Poilane 25052* (K), has young fruits, I found that it belongs to *S.foxworthianum*. Therefore, it is a new record of this species for Vietnam.

Eugeniadensifloravar.angustifolia Ridl. was described based on two specimens from Peninsular Malaysia, Peak (*Ridley s.n*.) and Kelantan (*Yapp s.n*.). *Ridley s.n*. (K) is available and is designated here as the lectotype.

### ﻿ Syzygium angkae

Taxon classificationPlantaeMyrtalesMyrtaceae

(Craib) Chantar. & J.Parn., Kew Bull. 48(3): 592. 1993.

B7A0D321-A543-5824-B18C-93A465E83F3F

Eugenia angkae Craib, Bull. Misc. Inform. Kew 1929: 115. 1929.

#### Type.

Thailand, Doi Inthanon (Doi Angka), 30 April 1921, *Kerr 5287* (lectotype, designated by Chantaranothai and Parnell 1994, p. 35: BK; isolectotypes: BM, K, TCD).

#### Distribution.

Myanmar, Thailand, Laos, Cambodia, Vietnam and China (Yunnan province, Menghai county, Xiding country, Mt. Dahei, Hesong, 2 May 2011, *Li-Jianwu 625* (HITBC no. 136852, QBG no. 64320, fruiting specimen).

#### Ecology.

In evergreen broad-leaf forest, ca. 1,960 m alt.

#### Notes.

*Syzygiumangkae* is characterized by having axillary or terminal inflorescences ca. 2 cm long and numerous secondary veins. It is found on mountains at an altitude of at least 1,500 m or more. The unidentified fruiting specimen at QBG belongs to *S.angkae*. The distribution of the species was mainly in Myanmar, Thailand, Laos, and Vietnam; it is now extended to China.

### ﻿ Syzygium thorelii

Taxon classificationPlantaeMyrtalesMyrtaceae

(Gagnep.) Merr. & L.M.Perry, J. Arnold Arbor. 19: 107. 1938.

B6C6BDDB-4253-58C6-A79A-E3DCE9C0880D

Eugenia thorelii Gagnep. in Lecomte., Notul. Syst. (Paris) 3: 333. 1918.

#### Type.

Thailand, Ubon Ratchathani, Kemmarat, *Thorel 3010* (lectotype, designated by Soh and Parnell 2015, p. 261: P [P00589178]; isolectotypes: A [A00069448], P [P00589179], K [K000276196]).

#### Distribution.

Thailand, Laos, Cambodia, Vietnam, China (Yunnan Province, Mengla County, Mt. Gongbeng, Luosuo River Estuary, 500 m alt., 3 Jan. 2011, *Li-Jianwu 239* (HITBG, no. 135842, QBG).

#### Ecology.

Open rock crevices in the Mekong River, partly submerged at high water or in sandy soils along the side of the river.

#### Notes.

*Syzygiumthorelii* is distinctive in having an obtuse or acute leaf apex and alternate leaves in the lower parts of the plant. A previously unidentified specimen seen at QBG undoubtedly belongs to *S.thorelii* and is a new record for China. The Chinese specimen extends the range of the species from Thailand, Laos, Cambodia and Vietnam to Yunnan.

##### ﻿New synonymy

### ﻿ Syzygium densinervium

Taxon classificationPlantaeMyrtalesMyrtaceae

(Merr.) Merr., Phillip. J. Sci. 79: 387. 1951.

A49E81EB-9669-5332-A5C4-372538C4907B

Eugenia densinevia Merr., Philipp. J. Sci. 1(Suppl.): 105. 1905. Type: Philippines, Luzon, Laguna province, Los Banos, Mt. Maquiling, Jun. 1917, *Elmer 18011*, (holotype K [K000800201]).
Eugenia silvestrei Elm, Leafl. Philipp. Bot. 8: 3095. 1919. Type: Philippines, Luzon Island, Los Baños, Mount Maquiling, July 1917, *Elmer 18011* (holotype A [A00069784]).
Syzygium densinervium var. insulare C.E.Chang, Bull. Taiwan Prov. Pingtung Inst. Agri. 5: 52. 1964. Type: Taiwan, Botel Tobago, 17 Apr. 1962, *Chang 2846*, (isotype L [L0009615]), syn. nov.

#### Type.

Based on *Eugeniadensinevia* Merr.

#### Distribution.

Philippines (Luzon) and Taiwan (South Cape).

#### Ecology.

In evergreen forest, mountain slope.

#### Notes.

*Syzygiumdensinervium* resembles *S.fastigiatum* (Blume) Merr. & L.M.Perry in having paniculate inflorescence, funnel-shaped hypanthium and persistent bracts and bracteoles. It differs by its rugulose hypanthial cup. I have examined an unidentified specimen, *Henry 1998* at K [K001003761] and found that it belongs to *S.densinervium*. I have also examined the isotype type of S.densinerviumvar.insulare from Taiwan and then placed it under *S.densinervium*. This species is distributed in the Philippines and Taiwan.

#### Specimens examined.

Taiwan, Botel Tobago, 7 Feb. 1980, *Chang 14691* (KYO); ibid., 6 Sept. 1980, *Chang 14694* (KYO).

### ﻿ Syzygium pellucidum

Taxon classificationPlantaeMyrtalesMyrtaceae

(Duthie) N.P.Balakr., Bull. Bot. Surv. India 22(1–4): 14. 1982.

D59D6012-8E69-51E2-80D4-CFD7F95DF5C1

Eugenia pellucida Duthie, Fl. Brit. India 25(4): 485. 1878. Type: Tenasserim & Andamans, *Helfer 2406* (lectotype, designated here: K [K000821333]; isolectotype A [GH00069438].
Eugenia pellucida var. contracta Wall. ex Duthie, Fl. Brit. India 25(4): 485. 1878. —Syzygiumcontractum Wall., *nom.nud*., non Eugeniacontracta Poir., 1828. Type: Myanmar, Ataran [Attran] river, 1827, *Wallich 3602* (lectotype, designated here: K-W [K001119797]; isolectotypes: K [K000821331, K000821332], A [A01143296]).
Eugenia ripicola Craib, Bull. Misc. Inform. Kew 1915(10): 428. 1915.— Syzygiumripicola (Craib) Merr. & L.M.Perry, Britt. 4: 127. 1941. Type: Thailand, Mae [Mê] Ping Rapids, Keng Soi, 16 Mar. 1913, *Kerr 2944* (lectotype, designated by Chantaranothai and Parnell 1994, p. 104: ABD; isolectotypes: BM [BM000944095], E [E00284095], K [K000800078]), syn. nov.
Eugenia cochinchinensis Gagnep. in Lecomte, Notul. Syst. (Paris) 3: 324. 1918.— Syzygiumcochinchinense (Gagnep.) Merr. & L.M.Perry, J. Arnold Arb. 19:107. 1938. Type: Cambodia, Kompong Speu, Samroang Tong, April 1870, *Pierre 527* (lectotype, designated by Soh and Parnell 2015, p. 254: P [P00589286]; isolectotypes: P [P00589287, P00589288, P00589350], syn. nov.
Eugenia eburnea Gagnep. in Lecomte, Notul. Syst. (Paris) 3: 324. 1918.— Syzygiumeburneum (Gagnep.) Merr. & L.M.Perry, J. Arnold Arb. 19:107. 1938. Type: Cambodia, plain of Pen-lovier, May 1870, *Pierre 991* (lectotype, designated by Soh and Parnell 2015, p. 255: P [P00589209]; isolectotypes: P [P00589210, P00589211], K [K000276209], E [E00284602], syn. nov.

#### Type.

Based on *Eugeniapellucida* Duthie

#### Distribution.

India, Myanmar, Thailand, Vietnam.

#### Ecology.

Along rivers or streams.

#### Notes.

Based on three collections, *Helfer 2406* (K000821333, GH00069438), *Helfer 2407* and *Kurz s.n.*, the last two have not been seen. Therefore, K000821333 is designated here as the lectotype of *E.pellucida.* The original description of E.pellucidavar.contracta is based on *Wallich 3602* which has four sheets. The specimen, K001119797 is designated here to be the lectotype because it has more leaves and inflorescences. *Syzygiumpellucidum* was considered to be endemic to Myanmar (POWO 2023) but its distribution is now extended to Thailand, Laos and Vietnam.

### ﻿ Syzygium prainianum

Taxon classificationPlantaeMyrtalesMyrtaceae

(King) Chantar. & J.Parn., Kew Bull. 48: 608. 1993.

A7934A2B-80CA-5F70-BC3B-035EFC2FB6E5

Eugenia prainiana King, J. Asiat. Soc. Bengal, Pt. 2, Nat. Hist. 70(1): 116. 1901. Type: Peninsular Malaysia, Perak, Blanta Mabok, Apr. 1890, *Wray 3990* (lectotype, designated by Chantaranothai and Parnell 1994, p. 97: K).
Syzygium prainianum subsp. minor Chantar. & J.Parn., Kew Bull. 48: 608. 1993. Type: Thailand, Phangnga, Khao (Kao) Kata Kwam, 9 Mar. 1930, *Kerr 18481* (holotype BM, isotypes BKF, K [K001007999]), syn. nov.

#### Type.

Based on *Eugeniaprainiana* King.

#### Distribution.

Thailand, Peninsular Malaysia, Borneo.

#### Ecology.

In evergreen forest, 900 m alt.

#### Notes.

Chantaranothai and Parnell (1993) proposed subsp. minor for Thai material with a slightly shorter hypanthial cup (3–4 mm long vs 5 mm long for the typical variety), stamens and style. After examination of *Gardner & Chamchumroon ST2467* K [001007729], a second new specimen collected from Trang, Thailand, I found that these characteristics are variable. Therefore, subsp. minor is here placed into synonymy of *S.prainianum*. This species is uncommon in Thailand but widespread in Peninsular Malaysia and Borneo.

#### Specimens examined.

Trang, Yanta Khao, Khao Banthat Wildlife Sanctuary, valley above Sai Rung Waterfall, Camp 2, 800 m alt., 14 Mar. 2006, *Gardner & Chamchumroon ST2467* (K).

## Supplementary Material

XML Treatment for Syzygium kampotense

XML Treatment for Syzygium cerasiforme

XML Treatment for Syzygium foxworthianum

XML Treatment for Syzygium angkae

XML Treatment for Syzygium thorelii

XML Treatment for Syzygium densinervium

XML Treatment for Syzygium pellucidum

XML Treatment for Syzygium prainianum

## ﻿Additional information

### Author ORCIDs

Pranom Chantaranothai https://orcid.org/0000-0002-5065-6169

### Data availability

All of the data that support the findings of this study are available in the main text.

## References

[B1] Ashton PS (2011) Myrtaceae*s.l.* In: Soepadmo E, Saw LG, Chung RCK, Kiew R (Eds) Tree flora of Sabah and Sarawak (Vol. 7). Sabah Forestry Department, Forest Research Institute Malaysia (FRIM), Sarawak Forestry Department, Malaysia, 87–423. 10.26525/TFSS7002

[B2] Biffin E Craven LA Crisp MD Gadek PA (2006) Molecular systematics of *Syzygium* and allied genera (Myrtaceae): Evidence from the chloroplast genome. Taxon 55(1): 79–94. 10.2307/25065530

[B3] Chantaranothai P (2014) Three new records of *Syzygium* Gaertn. in Thailand and lectotypification of 19 taxa of *Eugenia* L. (Myrtaceae). Thai Forest Bulletin. Botany 42: 75–80.

[B4] Chantaranothai P Parnell JAN (1993) New Taxa and Combinations in *Cleistocalyx* and *Syzygium* (Myrtaceae) in Thailand. Kew Bulletin 48(3): 589–610. 10.2307/4118723

[B5] Chantaranothai P Parnell JAN (1994) A revision of *Acmena*, *Cleistocalyx*, *Eugenia**s.s.* and *Syzygium* (Myrtaceae) in Thailand. Thai Forest Bulletin. Botany 21: 1–123.

[B6] Chantaranothai P Suksathan P Wongnak M (2016) *Syzygiumsirindhorniae* (Myrtaceae), a new species from Thailand. Phytotaxa 289(2): 193–196. 10.11646/phytotaxa.289.2.10

[B7] Chen J Craven LA (2007) Myrtaceae. In: Wu ZY Raven PH Hong DY (Eds) Flora of China (Vol.13). Science Press, Beijing, and Missouri Botanical Garden Press, St. Louis, 321–359.

[B8] Craven LA Biffin E (2010) An infrageneric classification of *Syzygium* (Myrtaceae). Blumea 55(1): 94–99. 10.3767/000651910X499303

[B9] Elmer ADE (1912) Notes and descriptions of *Eugenia.* Leaflets of the Philippine Botany 4: 1399–1444.

[B10] Hatt S Low YW Burslem DFRP Middleton DJ Biffin E Maurin O Lucas EJ (2023) A morphological analysis of *Syzygium*, with a focus on fibre bundles and description of a new subgenus. Botanical Journal of the Linnean Society 20(1): 1–7. 10.1093/botlinnean/boac065

[B11] IUCN (2012) IUCN Red List Categories and Criteria: Version 3.1 (2^nd^ edn). Gland and Cambridge, 32 pp.

[B12] Merrill ED Perry LM (1939) The myrtaceous genus *Syzygium* Gaertner in Borneo. Memoirs of the American Academy of Arts and Sciences 18(3): 135–202. 10.2307/25058505

[B13] Parnell JAN Chantaranothai P (2002) Myrtaceae. In: Santisuk T Larsen K (Eds) Flora of Thailand (Vol.7). The Forest Herbarium, Royal Forest Department, Bangkok, 778–914.

[B14] Poiret JLM (1828) *Eugeniacontracta*. In: Lamarck JBAPM, Poitet JLM (Eds) Encyclopédie méthodique. Botanique. Supplement. A. Paris, Chez H. Agasse, Impimeur- Libraire, 125 pp.

[B15] POWO (2023) Plants of the World Online. Facilitated by the Royal Botanic Gardens, Kew. Published on the Internet. http://www.plantsoftheworldonline.org/ [Retrieved 13/05/2023]

[B16] Soh WK Parnell JAN (2015) A revision of *Syzygium* Gaertn. (Myrtaceae) in Indochina (Cambodia, Laos and Vietnam). Adansonia 37(2): 179–275. 10.5252/a2015n2a1

[B17] Tagane S Van-son Dang Souladeth P Nagamasu H Toyama H Naiki A Tran H Yang Ceng-Jui Prajaksood A Yahara T (2018) Five new species of *Syzygium* (Myrtaceae) from Indochina and Thailand. Phytotaxa 375(4): 247–260. 10.11646/phytotaxa.375.4.1

[B18] Tagane S Toyama H Chhang P Nagamasu H Yahara T (2015) Flora of Bokor National Park, Cambodia I: Thirteen new species and one change in status. Acta Phytotaxonomica et Geobotanica 66(2): 95–135.

[B19] Thiers BM (updated continuously) Index Herbariorum. https://sweetgum.nybg.org/science/ih/

